# A fractional Fourier transform analysis of the scattering of ultrasonic waves

**DOI:** 10.1098/rspa.2014.0958

**Published:** 2015-03-08

**Authors:** Katherine M.M. Tant, Anthony J. Mulholland, Matthias Langer, Anthony Gachagan

**Affiliations:** 1 Department of Mathematics and Statistics, University of Strathclyde, Glasgow, UK; 2 Centre for Ultrasonic Engineering, University of Strathclyde, Glasgow, UK

**Keywords:** ultrasonics, non-destructive testing, inverse problems, scattering theory

## Abstract

Many safety critical structures, such as those found in nuclear plants, oil pipelines and in the aerospace industry, rely on key components that are constructed from heterogeneous materials. Ultrasonic non-destructive testing (NDT) uses high-frequency mechanical waves to inspect these parts, ensuring they operate reliably without compromising their integrity. It is possible to employ mathematical models to develop a deeper understanding of the acquired ultrasonic data and enhance defect imaging algorithms. In this paper, a model for the scattering of ultrasonic waves by a crack is derived in the time–frequency domain. The fractional Fourier transform (FrFT) is applied to an inhomogeneous wave equation where the forcing function is prescribed as a linear chirp, modulated by a Gaussian envelope. The homogeneous solution is found via the Born approximation which encapsulates information regarding the flaw geometry. The inhomogeneous solution is obtained via the inverse Fourier transform of a Gaussian-windowed linear chirp excitation. It is observed that, although the scattering profile of the flaw does not change, it is amplified. Thus, the theory demonstrates the enhanced signal-to-noise ratio permitted by the use of coded excitation, as well as establishing a time–frequency domain framework to assist in flaw identification and classification.

## 1. Introduction

Non-destructive testing (NDT) is an umbrella term for a wide and varied group of analysis techniques used to evaluate and characterize materials non-invasively [1]. Of particular interest to this paper are ultrasonic phased array systems, which have become increasingly popular as tools for flaw detection and characterization within the NDT industry. They provide improved resolution and coverage by transmitting and receiving ultrasound signals over multiple elements, which, when fired in predefined sequences, can provide increased control of beam directivity [2]. The existence and location of flaws are then deduced via images generated by post-processing the data captured by these arrays [3–5].

The examination of welds is of particular interest to the NDT community, given their role in safety critical structures in nuclear power plants, aeroengines, pipelines, etc. They are subject to cyclic loads and, as with any type of bond, constitute the weak point of the structure. Austenitic steel welds are notoriously difficult to inspect [1,6]. Owing to thermal effects as the weld is forming, a spatially heterogeneous structure is formed by local fluctuations in the crystal orientation. This complex internal geometry is highly scattering and results in low signal-to-noise ratio (SNR) levels, which can subsequently lead to the obscuration of defects. To combat this problem, two approaches are suggested and combined in this paper: the use of chirp excitations and the post-processing of the collected data in the time–frequency domain.

### (a) Chirp excitations

Coded excitations are an effective way of delivering large amounts of energy using relatively low acoustic pressure amplitudes. Inspiration for coded signal design can be drawn from bioacoustics; bats and dolphins use frequency-modulated sweeps to navigate and hunt [7,8]. The use of coded excitations in signal processing has been shown to improve SNR and lessen trade-offs between sample penetration and image resolution [9,10]. Chirps contain multiple frequencies which vary in time, typically in a linear or exponential manner. Their broad frequency content increases the likelihood of reaching the resonant frequency of a defect, in turn causing stronger vibrations and consequently improving the probability of detection. For the purposes of this work, a Gaussian-modulated linear chirp in time (*t*) of the form

**Figure 1.**
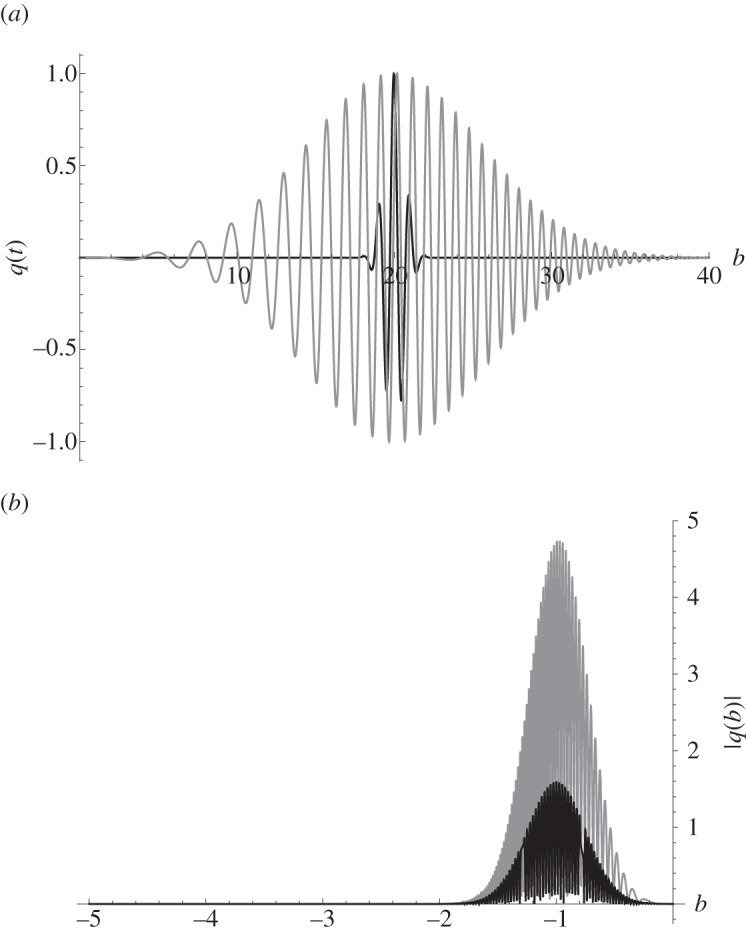
(*a*) A gated continuous sine wave with frequency *f*=1 MHz modulated by a Gaussian envelope with parameters *σ*=1 μs, *t*_1_= 20 μs (in black) and a Gaussian-modulated linear chirp with parameters *σ*=8 μs, *t*_1_=20 μs, *m*=0.22 MHz and *f*_1_= 0.1 MHz (in grey). Their respective Fourier transforms (power spectra) are shown in (*b*).

1.1 $$q(t)=\exp[-2\pi if_{1}(t+mt^{2})]\exp\left[-\frac{{\left(t-t_{1}\right)}^{2}}{\sigma^{2}}\right]$$

has been used, where *m* is the gradient of the chirp (the rate at which it sweeps through a prescribed range of frequencies), *f*_1_ is the initial frequency, *t*_1_ is the centre of the Gaussian envelope and *σ* is its standard deviation. By varying *f*_1_, *m* and *σ*, the bandwidth of the chirp can be altered. Setting *m*=0 Hz, the chirp reverts back to a time harmonic signal with frequency *f*_1_. For a fair comparison of the chirp with a continuous gated waveform (the typical signal emitted by the transducer), it is imperative both are optimized for the same transducer and hence use its full bandwidth. Figure 1 shows one such matched pair. In the time domain, the gated continuous sine wave (in black) spans a far shorter time interval than that of the Gaussian-modulated linear chirp (both signals have the same peak amplitude). Studying the plots of their respective Fourier transforms in figure 1*b*, it is apparent that they have similar −6 dB bandwidths (approx. 50%) but observe that the chirp contains a far greater amount of energy.

### (b) The fractional Fourier transform

An alternative approach to differentiating between flaw scattering and noise can be taken via the analysis of the collected data in the time–frequency domain. In an ideal case, a received ultrasound signal would exhibit signs of scattering at the time interval pertaining to the location of the flaw, facilitating detection and subsequent characterization. However, in practice, scattering by the microstructure of the host media can dominate the signal. To improve identification of defects it is suggested that the scattered signals are analysed for their frequency content; the frequency spectrum of the wave scattered by a flaw should be different from that scattered by a heterogeneity. However, time information must be retained in order to locate the flaw. This can be achieved via (i) the time windowed Fourier transform [11], where the discrete Fourier transform is applied to short time intervals allowing the frequency content at that specific time to be analysed independently of the rest of the signal, or (ii) the fractional Fourier transform (FrFT) [12–14], which enables continuous movement between the time and frequency domains, allowing the simultaneous retention of both frequency and time domain information. In this work, the FrFT is employed to analyse the benefits of chirp excitation over gated continuous wave excitation.

As a generalization of the ordinary Fourier transform, the FrFT is more flexible in its applications and hence of potential interest to any area in which the Fourier transform is frequently implemented. Its main advantage is that it allows continuous movement between the time and frequency domains, retaining information from each, thus presenting an alternative to using the time windowed Fourier transform. There exist several conventions for defining the FrFT, each of which gives a slightly different physical interpretation. For the purposes of this work, the FrFT of order *a* is given as the linear integral transform [12]

1.2 $$F_{a}(u)\equiv \int_{-\infty }^{\infty }K_{a}(u,u^{\prime })f(u^{\prime })\;du^{\prime },$$

where

1.3 $$K_{a}(u,u^{\prime })\equiv \sqrt{1-i\cot\alpha }\exp[i\pi (\cot\alpha u^{2}-2\csc\alpha uu^{\prime }+\cot\alpha u^{\prime 2})]$$

and *α*=*aπ*/2. When *a* is an integer, it denotes the number of repeated applications of the ordinary Fourier transform. Hence, setting *a*=1 (and, consequently, *α*=*π*/2), equation (1.2]) simplifies to

1.4 $$F_{1}(u)\equiv \int_{-\infty }^{\infty }f(u^{\prime })\;e^{-i2\pi uu^{\prime }}\;du^{\prime },$$

the ordinary Fourier transform.

## 2. Solving the inhomogeneous wave equation in time–frequency space

To build a mathematical framework to allow for analysis of the scattering of an ultrasonic chirp by a flaw, the wave equation with a time-dependent forcing function, *q*(*t*), will be solved in the time–frequency space. Note that *q*(*t*) is spatially independent and hence acts like a body force, affecting the whole flaw domain simultaneously. To justify this, it is assumed that the length of the flaw is smaller than the wavelength. This is in keeping with the low-frequency assumption made in the Born approximation [15] which is used later. A second assumption inherent to the Born approximation is that the transmission and reception of waves takes place at a distance from the flaw which is much larger than the wavelength: the far-field assumption. To begin, consider the non-homogeneous wave equation

2.1 $$\frac{\partial^{2}}{\partial t^{2}}f(\text{x},t)-c^{2}\nabla^{2}f(\text{x},t)=q(t),$$

where $\text{x}\in R^{3}$, t∈R and *c* is the wave speed. We demand that the solution is bounded in time and space and satisfies the Sommerfeld radiation condition

2.2 $$\frac{\partial f}{\partial r}-ikf=O(r^{-1})\;\text{for}\;r=|\text{x}|\to \infty ,$$

guaranteeing that the waves are outgoing and decay sufficiently fast so there exist no sources at infinity. It is also assumed that an initial pressure amplitude of *h*_0_ is present at the ultrasonic array. This simplified case will of course cover the detection of an object in a fluid host but is also relevant to certain restricted classes within elastodynamics such as the propagation of horizontal shear waves in an isotropic solid. As mentioned above, the applications of interest will involve heterogeneous materials (with spatially dependent wave speeds) but this is a reasonable model with which to start this investigation and the methodology will extend naturally to these more general cases. By [12], the FrFT, taken with respect to time, of a derivative of a function is given by

2.3 $$F_{a}\left({\left[{\left(2\pi i\right)}^{-1}\frac{d}{du}\right]}^{n}f(u)\right)={\left[u\sin\alpha +\cos\alpha {\left(2\pi i\right)}^{-1}\frac{d}{du}\right]}^{n}f_{a}(u).$$

Hence, taking the FrFT of every term in equation (2.1) gives

2.4 $$\begin{array}{rl} & {\left(2\pi i\right)}^{2}\sin^{2}\alpha u^{2}f_{a}(\text{x},u)+4\pi i\sin\alpha \cos\alpha u\frac{\partial }{\partial u}f_{a}(\text{x},u)\\ & \;+\cos^{2}\alpha \frac{\partial^{2}}{\partial u^{2}}f_{a}(\text{x},u)=c^{2}\nabla^{2}f_{a}(\text{x},u)+q_{a}(u), \end{array}$$

the non-homogeneous wave equation in time–frequency space.

### (a) The homogeneous solution

To solve equation (2.4), we start by finding the solution to the homogeneous differential equation via separation of variables. The solution is written in product form

2.5 $$f_{a}^{h}(\text{x},u)=h(\text{x})g_{a}(u)$$

and substituted into the homogeneous wave equation to give

2.6 $${\left(2\pi i\right)}^{2}\sin^{2}\alpha u^{2}+4\pi i\sin\alpha \cos\alpha u\frac{g_{a}^{\prime }(u)}{g_{a}(u)}+\cos^{2}\alpha \frac{g_{a}^{\prime \prime }(u)}{g_{a}(u)}=c^{2}\frac{\nabla^{2}h(\text{x})}{h(\text{x})}=-b^{2},$$

for some b∈R. This can then be separated into two equations

2.7 $$\left(-4\pi^{2}\sin^{2}\alpha u^{2}+i4\pi \sin\alpha \cos\alpha u\frac{d}{du}+\cos^{2}\alpha \frac{d^{2}}{du^{2}}\right)g_{a}(u)=-b^{2}g_{a}(u)$$

and

2.8 $$c^{2}\nabla^{2}h(\text{x})=-b^{2}h(\text{x}),$$

from which the temporal and spatial components of the homogeneous solution can be derived. To solve equation (2.7), a chirp-like ansatz of the form

2.9 $$g_{a}(u)=\exp[\gamma u^{2}+\beta u]$$

is chosen. Substituting this into equation (2.7) gives

2.10 $$\begin{array}{r} {}[-(4\pi^{2}\sin^{2}\alpha \;u^{2}-b^{2})+4\pi i\sin\alpha \cos\alpha u(2\gamma u+\beta )+\cos^{2}\alpha (2\gamma +{\left(2\gamma u+\beta \right)}^{2})]\;e^{\gamma u^{2}+\beta u}=0, \end{array}$$

that is,

2.11 $$\begin{array}{rl} & \left[u^{2}(4\gamma^{2}\cos^{2}\alpha +8\pi i\gamma \sin\alpha \cos\alpha -4\pi^{2}\sin^{2}\alpha )+u(4\gamma \beta \cos^{2}\alpha +4\pi i\beta \sin\alpha \cos\alpha \right)\\ & \;+(2\cos^{2}\alpha \gamma +\beta^{2}\cos^{2}\alpha +b^{2})]=0. \end{array}$$

Equating coefficients of powers of *u*, one can calculate *γ* and *β* as

2.12 $$\gamma =-\pi i\tan\alpha \;\text{and}\;\beta =\pm \sqrt{-b^{2}\sec^{2}\alpha +2\pi i\tan\alpha },$$

where the square root is taken so as the real part is positive. Hence

2.13 $$\begin{array}{rl} g_{a}(u) & =d_{1}\exp\left[u(-\pi i\tan\alpha u-\sqrt{-b^{2}\sec^{2}\alpha +2\pi i\tan\alpha })\right]\\ & \;+d_{2}\exp\left[u(-\pi i\tan\alpha u+\sqrt{-b^{2}\sec^{2}\alpha +2\pi i\tan\alpha })\right]. \end{array}$$

However, to ensure that the solution is bounded in *u*, *d*_2_ is set equal to zero.

Turning our attention now to the spatially dependent component of the homogeneous solution, equation (2.8) can be recognized as the homogeneous Helmholtz equation

2.14 $$\nabla^{2}h(\text{x})+{\hat{k}}^{2}h(\text{x})=0,$$

where $\hat{k}=b/c$ (*c* is the plane wave speed and *b* is analogous to the circular frequency). An explicit approximation of the scattered wave at a specified *b*, *h*(**x**,*b*), is derived via the Born approximation [15], giving

2.15 $$\begin{array}{r} h(\text{y},b)=h_{0}\frac{e^{i\hat{k}r_{s}}}{r_{s}}\frac{a_{1}a_{2}a_{3}[\gamma_{\lambda }-\gamma_{\rho }({\text{e}}_{i}\cdot {\text{e}}_{s})]}{|{\text{e}}_{i}-{\text{e}}_{s}{|}^{2}r_{e}^{2}}\left[\frac{\sin(\hat{k}|{\text{e}}_{i}-{\text{e}}_{s}|r_{e})-\hat{k}|{\text{e}}_{i}-{\text{e}}_{s}|r_{e}\cos(\hat{k}|{\text{e}}_{i}-{\text{e}}_{s}|r_{e})}{\hat{k}|{\text{e}}_{i}-{\text{e}}_{s}|r_{e}}\right], \end{array}$$

where *a*_1_, *a*_2_ and *a*_3_ represent the flaw dimensions (the flaw is modelled as an ellipsoid), *γ*_*ρ*_=1−*ρ*_0_/*ρ*_1_ (*ρ*_0_ and *ρ*_1_ are the material densities of the host and flaw materials, respectively), *γ*_λ_=1−λ_0_/λ_1_ (λ_0_ and λ_1_ are the bulk moduli of the host and flaw materials, respectively), *r*_*s*_ is the distance of the flaw from the array, *h*_0_ is the initial pressure amplitude, **e**_*i*_ and **e**_*s*_ are unit vectors in the incident and scattered wave directions, respectively, and *r*_*e*_ is the effective radius of the flaw, given by

2.16 $$r_{e}=\sqrt{a_{1}^{2}{\left({\text{e}}_{q}\cdot {\text{u}}_{1}\right)}^{2}+a_{2}^{2}{\left({\text{e}}_{q}\cdot {\text{u}}_{2}\right)}^{2}+a_{3}^{2}{\left({\text{e}}_{q}\cdot {\text{u}}_{3}\right)}^{2}},$$

where

2.17 $${\text{e}}_{q}=\frac{{\text{e}}_{i}-{\text{e}}_{s}}{|{\text{e}}_{i}-{\text{e}}_{s}|}$$

and the unit vectors **u**_1_, **u**_2_ and **u**_3_ lie along the axes of the flaw. Thus, a solution to the homogeneous equation can be written as

2.18 $$\begin{array}{rl} f_{a}^{h}(\text{x},u,b) & =d_{1}\exp\left[u(-i\pi \tan\alpha u-\sqrt{-b^{2}\sec^{2}\alpha +i2\pi \tan\alpha })\right]\\ & \;\times h_{0}\frac{e^{i\hat{k}r_{s}}}{r_{s}}\frac{a_{1}a_{2}a_{3}[\gamma_{\lambda }-\gamma_{\rho }({\text{e}}_{i}\cdot {\text{e}}_{s})]}{|{\text{e}}_{i}-{\text{e}}_{s}{|}^{2}r_{e}^{2}}\\ & \;\times \left[\frac{\sin(\hat{k}|{\text{e}}_{i}-{\text{e}}_{s}|r_{e})-\hat{k}|{\text{e}}_{i}-{\text{e}}_{s}|r_{e}\cos(\hat{k}|{\text{e}}_{i}-{\text{e}}_{s}|r_{e})}{\hat{k}|{\text{e}}_{i}-{\text{e}}_{s}|r_{e}}\right]. \end{array}$$

Because any superposition of solutions of a linear, homogeneous PDE is again a solution, the dependence on *b* can be removed by integrating over *b*

2.19 $$\begin{array}{rl} f_{a}^{h}(\text{x},u) & =\int_{-\infty }^{\infty }d_{1}\exp\left[u(-i\pi \tan\alpha u-\sqrt{-b^{2}\sec^{2}\alpha +i2\pi \tan\alpha })\right]\\ & \;\times h_{0}\frac{e^{i\hat{k}r_{s}}}{r_{s}}\frac{a_{1}a_{2}a_{3}[\gamma_{\lambda }-\gamma_{\rho }({\text{e}}_{i}\cdot {\text{e}}_{s})]}{|{\text{e}}_{i}-{\text{e}}_{s}{|}^{2}r_{e}^{2}}\\ & \;\times \left[\frac{\sin(\hat{k}|{\text{e}}_{i}-{\text{e}}_{s}|r_{e})-\hat{k}|{\text{e}}_{i}-{\text{e}}_{s}|r_{e}\cos(\hat{k}|{\text{e}}_{i}-{\text{e}}_{s}|r_{e})}{\hat{k}|{\text{e}}_{i}-{\text{e}}_{s}|r_{e}}\right]db. \end{array}$$

However, to ensure that this integral is finite, *d*_1_ must be chosen as some function of *b* with which to bound the solution. For this work, the function

2.20 $$d_{1}(b)=A\exp\left[\frac{-{\left(b-b_{1}\right)}^{2}}{\sigma^{\prime 2}}\right]$$

has been chosen, where *A*, *b*_1_ and *σ*′ are chosen to mimic the Gaussian envelope observed in the plot of the power spectrum of the forcing function (figure 1*b*). Thus, the solution is effectively summed over the range of frequencies received by the transducer array.

### (b) The inhomogeneous solution

To find a time-dependent inhomogeneous solution $f_{a}^{p}(u)$ to equation (2.1), an ansatz of the form

2.21 $$f_{a}^{p}(u)=\int_{-\infty }^{\infty }v_{a}(b)s_{a}(u,b)\;db$$

is chosen, where

2.22 $$s_{a}(u,b)=\sqrt{1+i\tan\alpha }\exp[-i\pi (u^{2}\tan\alpha +2ub\sec\alpha +b^{2}\tan\alpha )].$$

This form is chosen since, using the identities tan⁡α=−cot⁡(α+π/2) and sec⁡α=csc⁡(α+π/2), one can rewrite *s*_*a*_(*u*,*b*) as

2.23 $$\begin{array}{rl} s_{a}(u,b) & =\sqrt{1-\cot\left(\alpha +\frac{\pi }{2}\right)}\\ & \;\times \exp\left[\pi i\left(u^{2}\cot\left(\alpha +\frac{\pi }{2}\right)-2ub\csc\left(\alpha +\frac{\pi }{2}\right)+b^{2}\cot\left(\alpha +\frac{\pi }{2}\right)\right)\right]\\ & =K_{a+1}(u,b), \end{array}$$

the kernel of the FrFT of order *a*+1. The derivatives of *s*_*a*_(*u*,*b*) are given by

2.24 $$\begin{array}{rl} \frac{\partial }{\partial u}s_{a}(u,b) & =\sqrt{1+i\tan\alpha }(-2\pi iu\tan\alpha -2\pi ib\sec\alpha )\\ & \;\times \exp[-\pi i(u^{2}\tan\alpha +2ub\sec\alpha +b^{2}\tan\alpha )] \end{array}$$

and

2.25 $$\begin{array}{rl} \frac{\partial^{2}}{\partial u^{2}}s_{a}(u,b) & =\sqrt{1+i\tan\alpha }[{\left(-2\pi iu\tan\alpha -2\pi ib\sec\alpha \right)}^{2}-2\pi i\tan\alpha ]\\ & \;\times \exp[-\pi i(u^{2}\tan\alpha +2ub\sec\alpha +b^{2}\tan\alpha )]. \end{array}$$

Substituting these into equation (2.4) shows that the left-hand side can be written as

2.26 $$\begin{array}{rl} & {\left(2\pi i\right)}^{2}\sin^{2}\alpha u^{2}s_{a}(u,b)+i4\pi \sin\alpha \cos\alpha u\frac{\partial }{\partial u}s_{a}(u,b)+\cos^{2}\alpha \frac{\partial^{2}}{\partial u^{2}}s_{a}(u,b)\\ & \;=\sqrt{1+i\tan\alpha }\exp[-\pi i(u^{2}\tan\alpha +2ub\sec\alpha +b^{2}\tan\alpha )]\\ & \;\times [-4\pi^{2}\sin^{2}\alpha u^{2}+8\pi^{2}u^{2}\sin^{2}\alpha +8\pi^{2}ub\sin\alpha -2\pi i\sin\alpha \cos\alpha \\ & \;-4\pi^{2}u^{2}\sin^{2}\alpha -8\pi^{2}ub\sin\alpha -4\pi^{2}b^{2}]\\ & \;=\sqrt{1+i\tan\alpha }\exp[-\pi i(u^{2}\tan\alpha +2ub\sec\alpha +b^{2}\tan\alpha )](-4b^{2}\pi^{2}-\pi i\sin2\alpha )\\ & \;=s_{a}(u,b)(-4b^{2}\pi^{2}-\pi i\sin2\alpha ). \end{array}$$

Substituting equation (2.21) into equation (2.4) then gives

2.27 $$\int_{-\infty }^{\infty }(-4b^{2}\pi^{2}-\pi i\sin2\alpha )v_{a}(b)s_{a}(u,b)\;db=q_{a}(u).$$

Note that, when *u* lies along the *a*=0 axis, it is analogous to the original time domain signal. In the work below, *q*_0_(*u*) will be written as *q*(*t*) and *q*_1_(*u*) as *q*(*b*), to allow for easier physical interpretation. By letting $D(b)=(-4b^{2}\pi^{2}-i\pi \sin2\alpha )v_{a}(b)$ (and remembering *s*_*a*_(*u*,*b*)=*K*_*a*+1_(*u*,*b*)) equation (2.27) can be written as

2.28 $$F_{a+1}(D(b))=F_{a}(q(t)),$$

which holds for all orders *a*. Hence, by the index additivity rules it is shown that

2.29 $$F_{1}(D(b))=q(t).$$

By taking the FrFT of order *a*=−1 (analogous with the inverse Fourier transform) of both sides, it follows that

2.30 $$D(b)=\int_{-\infty }^{\infty }q(t)\;e^{i2\pi tb}\;dt,$$

and the unknown function *v*_*a*_(*b*) of the integral equation (2.27) can thus be found.

### (c) The inverse Fourier transform of a Gaussian-modulated linear chirp

An analytical expression for the inverse Fourier transform of a Gaussian-modulated linear chirp must now be derived. To evaluate equation (2.30), the chirp given by equation (1.1) must be rewritten in the form

2.31 $$q(t)=E_{0}\exp[-(p+ir)t^{2}-iw_{0}t],$$

where $E_{0}=\exp(-t_{1}^{2}/\sigma^{2})$, *p*=1/*σ*^2^, *r*=2*πf*_1_*m* and *w*_0_=2*πf*_1_+2*t*_1_*i*/*σ*^2^. Applying the inverse Fourier transform gives

2.32 $$D(b)=\int_{-\infty }^{\infty }E_{0}\exp[-((p+ir)t^{2}-i(2\pi b-w_{0})t)]\;dt.$$

The standard integral for a general quadratic exponent is calculated using

2.33 $$\int_{-\infty }^{\infty }\exp[-ax^{2}+bx+c]\;dx=\sqrt{\frac{\pi }{a}}\exp\left[\frac{b^{2}}{4a}+c\right],$$

assuming that the real part of *a* is positive. It follows that

2.34 $$D(b)=\frac{\sqrt{\pi }}{\sqrt{p+ir}}E_{0}\exp\left(\frac{-{\left(2\pi b-w_{0}\right)}^{2}}{4(p+ir)}\right),$$

and thus

2.35 $$v_{a}(b)=\frac{\sqrt{\pi }}{\sqrt{p+ir}}\frac{E_{0}}{\left(-4b^{2}\pi^{2}-i\pi \sin2\alpha \right)}\exp\left(\frac{-{\left(2\pi b-w_{0}\right)}^{2}}{4(p+ir)}\right).$$

Finally, by equations (2.22) and (2.35), the inhomogeneous solution of the wave equation in time–frequency space is given by

2.36 $$\begin{array}{rl} f_{a}^{p}(u) & =\frac{\sqrt{\pi }}{\sqrt{p+ir}}\int_{-\infty }^{\infty }\frac{E_{0}}{\left(-4b^{2}\pi^{2}-i\pi \sin2\alpha \right)}\exp\left(\frac{-{\left(2\pi b-w_{0}\right)}^{2}}{4(p+ir)}\right)\\ & \;\times \sqrt{1+i\tan\alpha }\exp(-i\pi (u^{2}\tan\alpha +2ub\sec\alpha +b^{2}\tan\alpha ))\;db. \end{array}$$

### (d) The general solution

Using equations (2.19) and (2.36), the general expression for the scattering from a flaw owing to the excitation by a linear chirp forcing function is given by

2.37 $$\begin{array}{rl} f_{a}(\text{x},u) & =\int_{-\infty }^{\infty }d_{1}(b)\exp\left[u(-i\pi \tan\alpha u-\sqrt{-b^{2}\sec^{2}\alpha +i2\pi \tan\alpha })\right]\\ & \;\times h_{0}\frac{e^{i\hat{k}r_{s}}}{r_{s}}\frac{a_{1}a_{2}a_{3}[\gamma_{\lambda }-\gamma_{\rho }({\text{e}}_{i}\cdot {\text{e}}_{s})]}{|{\text{e}}_{i}-{\text{e}}_{s}{|}^{2}r_{e}^{2}}\\ & \;\times \left[\frac{\sin(\hat{k}|{\text{e}}_{i}-{\text{e}}_{s}|r_{e})-\hat{k}|{\text{e}}_{i}-{\text{e}}_{s}|r_{e}\cos(\hat{k}|{\text{e}}_{i}-{\text{e}}_{s}|r_{e})}{\hat{k}|{\text{e}}_{i}-{\text{e}}_{s}|r_{e}}\right]\\ & \;+\frac{\sqrt{\pi }}{\sqrt{p+ir}}\frac{E_{0}}{\left(-4b^{2}\pi^{2}-i\pi \sin2\alpha \right)}\exp\left(\frac{-{\left(2\pi b-w_{0}\right)}^{2}}{4(p+ir)}\right)\\ & \;\times \sqrt{1+i\tan\alpha }\exp(-i\pi (u^{2}\tan\alpha +2ub\sec\alpha +b^{2}\tan\alpha ))\;db. \end{array}$$

Note that the spatial component of the solution, which gives rise to the scattering profile of the defect and encapsulates the geometry of the flaw, is amplified by the chirp excitation and this suggests that an increased SNR will result.

## 3. Comparison of a gated continuous wave excitation with a chirp excitation

A general solution to the inhomogeneous wave equation in the time–frequency domain with a Gaussian-modulated linear chirp forcing function has been derived. It can be observed that the additive inhomogeneous solution does not carry flaw shape information but does involve the chirp excitation parameters. Hence, to contrast the excitation by a chirp with that of a gated continuous wave, attention can be restricted to the inhomogeneous solution only.

**Figure 2.**
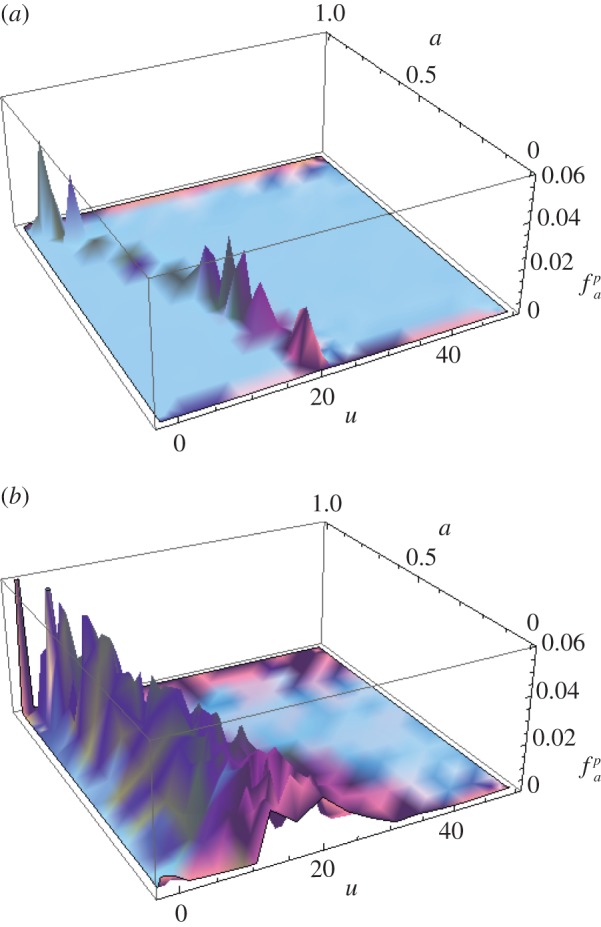
Three-dimensional surface plots of the inhomogeneous solution given by equation (2.36) with chirp parameters (*a*) *f*_1_=1 MHz, *m*= 0 MHz, *t*_1_=20 μs and *σ*=1 μs (gated continuous wave) and (*b*) *f*_1_=0.1 MHz, *m*=0.22 MHz, *t*_1_=20 μs and *σ*=8 μs (Gaussian-modulated linear chirp). (Online version in colour.)

**Figure 3.**
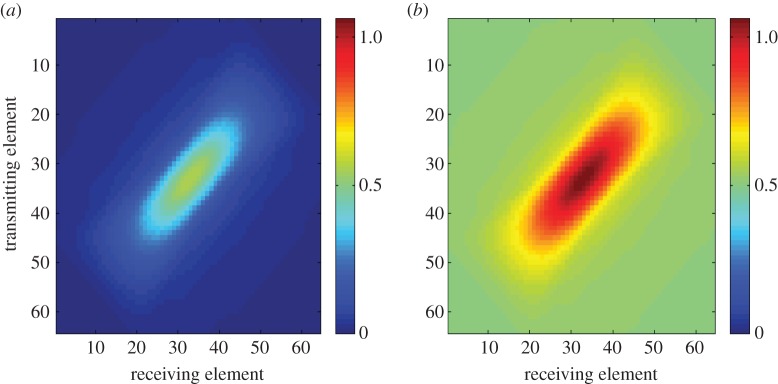
Sum of the scattering matrices as generated by the Born approximation arising from (*a*) gated continuous wave excitation and (*b*) linear chirp insonification. (Online version in colour.)

To compute the inhomogeneous solution numerically, the infinite integral over *b* is approximated by the integral over the bandwidth of the transducer. This is justified as it can be assumed that the integral is zero outside this interval (the received signal will not contain any frequencies outside of the bandwidth). This approach also resolves any issues around the singularity in the integrand when *b*=0 and *α*=0. The inhomogeneous solution is plotted as a time-order plot in figure 2 for the cases where the forcing function is set as (i) a gated continuous wave and (ii) a Gaussian-modulated linear chirp. Each point within the plot is the additional amplitude at that point in time–frequency space obtained by using the particular excitation. It can be seen that, since the linear chirp contains more energy, it provides a marked increase in the scattering amplitude over the entire time–frequency space. In the case of the gated continuous wave in figure 2*a*, some orders of the FrFT (for example *a*=0.55) offer no additive amplification at any point along *u*. To further demonstrate the benefits of excitation by the chirp, the corresponding frequency domain scattering matrices for a crack-like flaw (generated by the spatial component of the homogeneous solution over a range of transmit/receive directions, **e**_*i*_ and **e**_*s*_, which mimic inspection by a linear array) have been summed over the frequency range of the hypothetical transducer. The amplitudes along the *a*=1 axes in figure 2*a*,*b* are used to amplify the scattering matrices at the corresponding frequencies for the continuous gated wave excitation and the linear chirp excitation, respectively. The results are shown in figure 3. Geometrically, the scattering matrices are identical; however, it can be observed that the scattering profile of the flaw is amplified in the case of chirp insonification (figure 3*b*). It is clear that, if noise were present in the signal, the higher amplitudes exhibited in the case of chirp excitation would provide an increased SNR.

### (a) Choosing the optimal order *a*

**Figure 4.**
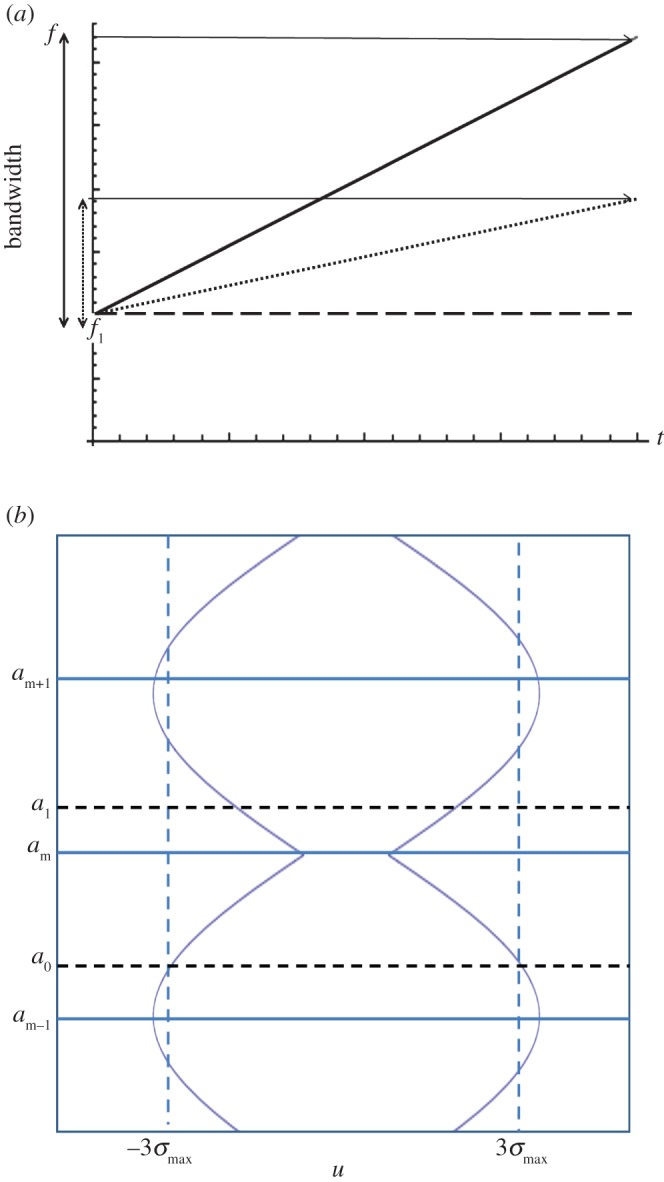
(*a*) Schematic demonstrating the increase in bandwidth with the increase of chirp rate *m*. The long-dashed line is representative of a single frequency pulse and its bandwidth is a delta function at that frequency. The dotted and solid lines represent linear chirps with increasing gradients which result in increased bandwidths. (*b*) Schematic demonstrating the change in *σ*_*a*_ in the fractional Fourier domain. (Online version in colour.)

**Figure 5.**
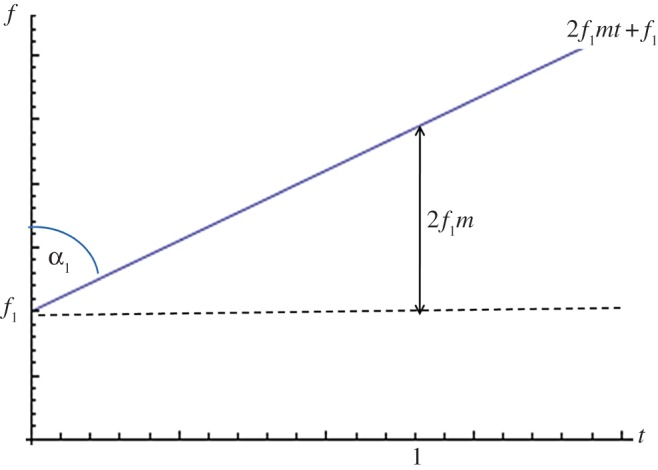
The geometrical interpretation of the optimal *α* at which to take the FrFT, dependent on *m* and *f*_1_. (Online version in colour.)

**Figure 6.**
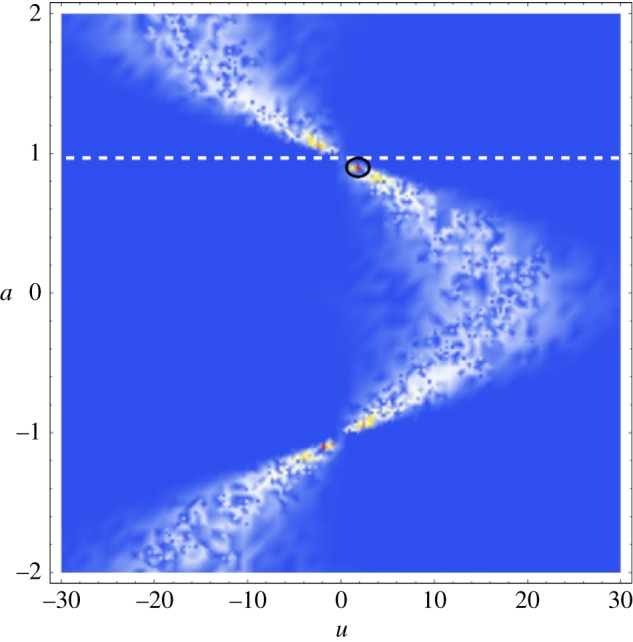
The inhomogeneous solution as given by equation (2.36), arising from linear chirp excitation with parameters *m*=0.22 MHz, *f*_1_= 0.1 MHz, *σ*=8 μs and *t*_1_=20 μs. The dashed line marks the order *a* at which the formula derived in [17] predicts the optimal value of *a* should occur. The predicted optimal *a* does not correspond to the order at which the maximum peak occurs (which is circled in black). (Online version in colour.)

It is shown in [16] that the FrFT of a Gaussian function has the form of a Gaussian for all orders *a*. The standard deviation of these Gaussian functions, *σ*_*a*_, varies in time–order space, with the narrowing of the function being synonymous with an increase in maximum amplitude. Hence, it can be concluded that the optimal value of *a* at which to employ the FrFT is the value at which the minimum *σ*_*a*_ occurs. As the chirp rate *m* increases, this maximum peak (which occurs at $\min\sigma_{a}$) moves further away from the frequency domain. This is explained schematically in figure 4. The long-dashed horizontal line in figure 4*a* represents a continuous wave (where *m*=0) and hence results in a single value on the frequency axis. As the gradient increases (see the dotted and solid lines), the breadth of the frequency spectrum increases. The curves in figure 4*b* demonstrate how the width of the Gaussian changes in the fractional Fourier domain (of course, the Gaussian is infinite but here the width is approximated by 6*σ*_*a*_ as 99.73% of the signal lies within this interval). The fractional order which exhibits the widest Gaussian is orthogonal to the order with the narrowest distribution (i.e. there is a difference of 1 between the orders at which these extremes occur). It then follows that, as the bandwidth of the chirp decreases, the order at which the narrowest Gaussian form arises approaches the frequency domain. Now, plotting the rate of frequency change (with respect to time) of the linear chirp results in the plot as seen in figure 5. The angle made with the frequency axis can thus be calculated as $\alpha =\tan^{-1}(\frac{1}{2}f_{1}m)$. This provides the optimum angle at which to take the FrFT [17] and translates to order $a=2\tan^{-1}(\frac{1}{2}f_{1}m)/\pi$. To assess the formula's success in predicting this order in regards to the inhomogeneous solution derived above, $f_{a}^{p}(u)$ (as defined in equation (2.36)) is plotted over orders −2≤*a*≤2 in figure 6. As the Gaussian function is not centred at zero, the plot appears skewed; however, the narrowing phenomena can still be observed. Owing to the low gradient of the chirp excitation, the location of the narrowest distribution approaches the frequency domain as predicted. The simple algebraic formula derived for the optimal order of the FrFT of a Gaussian-windowed linear chirp exhibits an error in application to the inhomogeneous solution and does not incorporate the maximum amplitude (which is circled in black). However, the error is small (within 0.1 of the order at which the maximum does occur) and the formula could potentially guide the implementation of the discrete FrFT [18], effectively reducing the neighbourhood (and, subsequently, the computational expense) over which the FrFT is taken. It is hoped that the general solution as derived above (equation (2.37)) will act as a basis for further work on improving the extraction of the optimal order *a* at which to implement the FrFT for signals which have encountered a defect and been subsequently scattered, thus eventually reducing the time–order space to one dimension for numerical implementations.

## 4. Conclusion

A wealth of information regarding the detection, imaging and sizing of flaws is contained within the scattering matrices as constructed by data arising from ultrasonic phased array inspections. Of course, the successful extraction of this information relies on the received data having a reasonable SNR. This paper examined the use of chirp excitation as a means of improving the SNR by increasing the amplitude of the recovered signal. A general solution to the inhomogeneous wave equation, and the subsequent scattering by a flaw, in the time–frequency domain, with a Gaussian-modulated linear chirp forcing function has been derived. This was achieved by taking the FrFT of the inhomogeneous wave equation and finding the homogeneous solution via separation of variables and the Born approximation. The inhomogeneous solution was obtained by choosing an ansatz that, once substituted into the inhomogeneous wave equation, resulted in a linear integral equation which could be solved by formulating a Fourier transform pair for a Gaussian-modulated linear chirp. Since the excitation parameters are exclusively contained in the additive term provided by the inhomogeneous solution, the comparison between gated continuous wave excitation and chirp excitation can be drawn by focusing solely on that term. It was plotted in a time–order plot for the cases where the forcing function was set as (i) a gated continuous wave and (ii) a Gaussian-modulated linear chirp and it was shown that, since the linear chirp contained more energy, there was a marked increase in the scattering amplitude. This was reinforced by plotting and comparing the corresponding scattering matrices for a chosen peak in the time–order plot, which further demonstrated the increased amplification provided by the chirp. Thus it is anticipated that an improved SNR will result when applied to experimental data.

Recent studies on the use of the FrFT with regard to chirp signals [16,17] suggest a potential methodology for studying the wave scattering problem considered in this paper. Indeed, the findings from that work were assessed for their applicability in this paper and, whilst the discrepancies were clear, they did provide a reasonable estimate of the optimal order at which to implement the FrFT. It is envisaged that the analytical formulation of the general solution in §2*d* will allow for improved extraction of the optimal *a* for the more complicated case of a chirp which has been scattered by a defect. The benefit in doing so would be the effective reduction of the time–frequency space to one dimension, thus enabling reduced computational cost in NDT applications.
